# CoDing Sequence Typer (CDST): a fast, simple, decentralized and interoperable solution for bacterial genomic typing and clustering

**DOI:** 10.1099/mgen.0.001518

**Published:** 2025-10-23

**Authors:** Menghan Li, Séamus Fanning, Zhemin Zhou, Li Bai

**Affiliations:** 1 National Health Commission Key Laboratory of Food Safety Risk Assessment, China National Center for Food Safety Risk Assessment, Beijing 100021, PR China; 2 UCD-Centre for Food Safety, School of Public Health, Physiotherapy & Sports Science, University College Dublin, Dublin D04 N2E5, Ireland; 3 The Second Affiliated Hospital of Soochow University, Cancer Institute, Suzhou Medical College, Soochow University, Suzhou 215127, PR China; 4 National Center of Technology Innovation for Biopharmaceuticals, Suzhou Biomedical Industry Innovation Center, Suzhou 215127, PR China; 5 National Key Laboratory of Intelligent Tracking and Forecasting for Infectious Diseases, National Institute for Communicable Disease Control and Prevention, Chinese Center for Disease Control and Prevention, Beijing, PR China; 6 Department of Nutrition and Food Hygiene, School of Population Medicine and Public Health, Chinese Academy of Medical Sciences & Peking Union Medical College, Beijing 100006, PR China

**Keywords:** bacteria genome typing, core-genome multilocus sequence typing (cgMLST), outbreak surveillance, *Salmonella enterica*

## Abstract

We present a fully decentralized MD5 hash-based framework that indexes predicted coding sequences (CDSs) and computes pairwise genomic distances without locus annotation or a central database. The method is implemented in the open-source CoDing Sequence Typer (CDST) pipeline, which delivers reproducible, privacy-preserving and computationally efficient bacterial typing. Applied to 1,961 complete *Salmonella enterica* genomes, CDST produced distance matrices that were highly concordant with core-genome multilocus sequence typing (cgMLST) and whole-genome MLST (wgMLST), core-genome SNP, Mash and Split Kmer analysis. In a 100-genome benchmark, CDST achieved ~8× faster runtimes than cg/wgMLST workflows and reduced storage to ~4% of the original assembly FASTA size. Unsupervised clustering evaluation identified three optimal resolution levels, *HC67 *(outbreak-level), *HC186* (lineage/serotype-level) and *HC441* (global structure-level), that align well with conventional typing schemes. These levels demonstrated high internal cohesion and external consistency with conventional typing schemes. Cross-species validation on *Listeria monocytogenes* and *Escherichia coli* genomes confirmed that the pipeline recovers species-specific population structures without parameter adjustment. Collectively, CDST provides a scalable and interoperable framework for bacterial population structure analysis, suitable for surveillance and outbreak investigations across laboratories. The CDST pipeline, along with all evaluation scripts, is openly available on GitHub at https://github.com/l1-mh/CDST.

**Impact Statement**

CoDing Sequence Typer removes the need for locus databases and whole-genome uploads, enabling privacy-preserving, cross-laboratory *Salmonella* surveillance. Its three hierarchical thresholds (*HC67/186/441*) directly mirror outbreak, lineage and population scales, providing an immediately deployable framework for public-health genomics.

## Data Summary

RefSeq accession numbers for all 1961 *Salmonella enterica* genomes are provided in Table S1. RefSeq accession numbers for *Listeria monocytogenes* and *Escherichia coli* genomes are provided in Table S2. The CoDing Sequence Typer source code (v.0.2.0), example JSON databases and evaluation scripts are available under a GNU General Public License v3.0 at https://github.com/l1-mh/CDST.

## Introduction

Accurate genomic typing is critical for the surveillance and investigation of bacterial pathogens. Among the available methods, core-genome multilocus sequence typing (cgMLST) and whole-genome multilocus sequence typing (wgMLST) have emerged as standard approaches for precise strain typing and epidemiological tracking [1]. In conventional workflows, sample sequences are uploaded to a central server where complete gene sequences are annotated and aligned against a reference database to determine allele types. This centralized strategy poses two main challenges: maintaining and updating a central repository imposes substantial computational and storage burdens; uploading entire genomes also raises data-privacy concerns, wherein uploading entire genomic sequences increases the risk of exposing sensitive genetic information.

To address these challenges, Zhong *et al*. and Deneke *et al*. separately proposed the distributed cgMLST method utilizing Message-Digest algorithm 5 (MD5) hash conversion [2,3]. This approach disperses the computational load to the user side, thereby reducing central server requirements and data exposure. Nonetheless, the method still relies on comparison with a reference database for locus identification, implying that full decentralization is not achieved.

Methods independent of predefined gene or loci annotations include core-genome SNP (cgSNP) analysis, Mash, Split Kmer analysis (SKA) and average nucleotide identity (ANI). cgSNP analysis allows for genome comparison by directly generating pairwise distances between sequences [4]. ANI is a widely used method that calculates pairwise distances between genome sequences based on their average sequence similarity using sequence alignment tools such as blast or MUMmer [5]. Mash is a method that compares genome sequences using MinHash sketches of *k-mers* [6]. SKA is another alignment-free approach that compares genome sequences by identifying and counting split *k-mers*, which are *k-mers* interrupted by a single base [7]. Although these methods are decentralized, they face limitations in data sharing and comparison: the high sensitivity of cgSNP makes it dependent on the initial reference dataset, ANI requires sequence alignment which is computationally intensive, while Mash and SKA are influenced by *k-mer* selection parameters. This dependence on input data and computational parameters poses challenges for inter-laboratory comparisons and data integration.

**Fig. 1.**
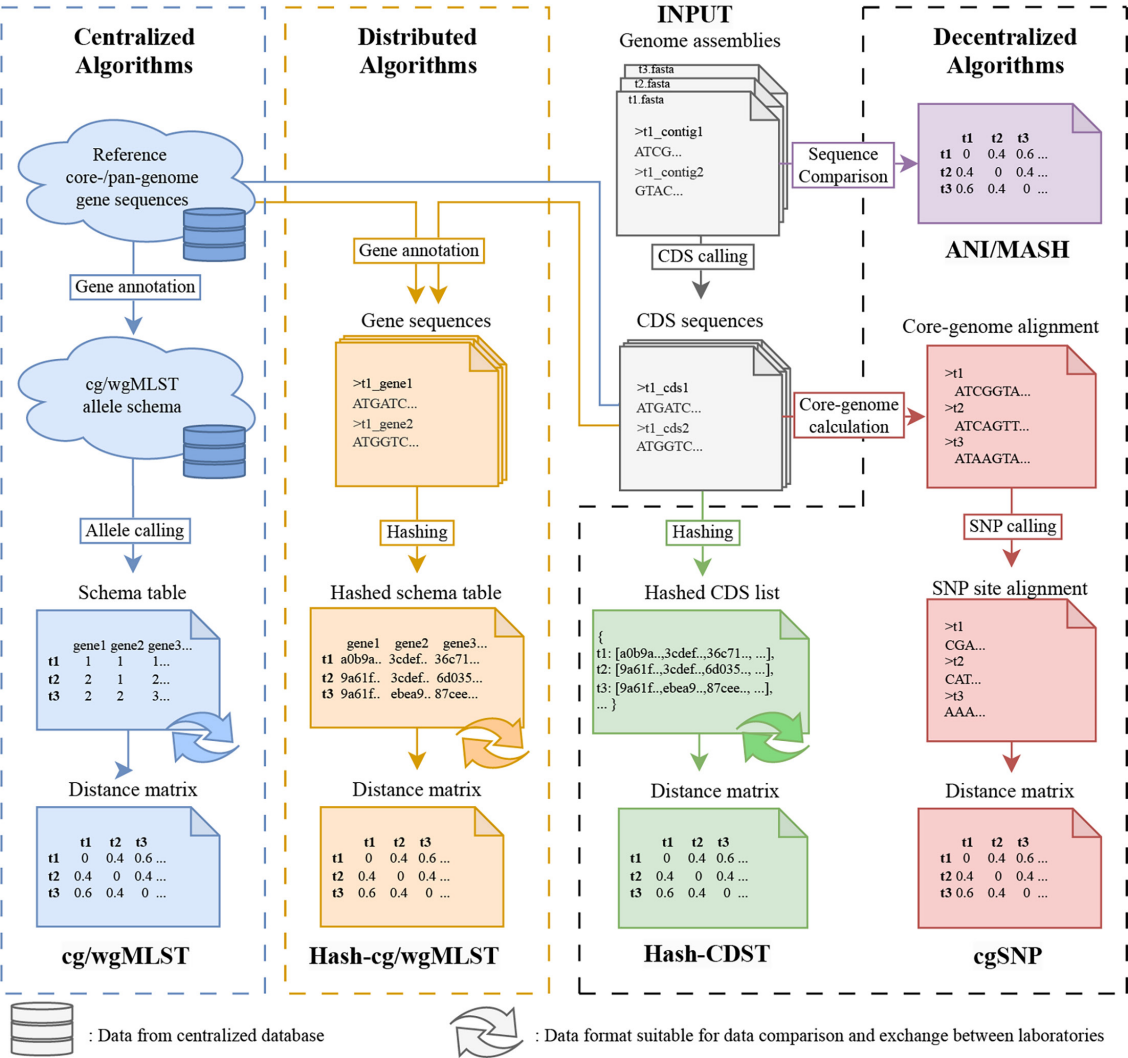
Comparison of genome typing and clustering workflows. The figure illustrates centralized, distributed and decentralized strategies for deriving pairwise distance matrices from input genome assemblies. Key steps involved in each method are shown. Data accessed by centralized reference databases and data suitable for inter-laboratory exchange are indicated with specific symbols.

Building on the strengths of existing genome typing methods, our study introduces a fully decentralized approach termed hash-based coding sequence typing, implemented in the CoDing Sequence Typer (CDST) tool. Unlike centralized methods such as cgMLST, which rely on a central reference database, and distributed methods like hash–cgMLST, which still require predefined loci for comparison, CDST achieves complete decentralization by performing direct MD5 hash mapping on predicted coding sequences (CDS) from genome assemblies. This approach eliminates the need for locus annotation, simplifying the workflow and significantly reducing computational and storage requirements. Furthermore, the irreversible nature of MD5 hashing enhances data privacy while enabling direct comparison and seamless integration of results across laboratories without dependency on shared reference databases. The conceptual framework and comparison of centralized, distributed and decentralized genome typing methods are illustrated in Fig. 1.

Therefore, we set out to (i) develop a locus-free, MD5 hash-based typing framework; (ii) benchmark its concordance with cgMLST, wgMLST, cgSNP, Mash and SKA across 1,961 *Salmonella enterica* genomes; and (iii) define practical clustering thresholds corresponding to outbreak, lineage and population scales.

## Methods

### Algorithm overview

The CDST pipeline bypasses the traditional locus allele alignment process by predicting CDS directly from genome assemblies and then applying MD5 hash mapping to each CDS sequence. Given that the MD5 algorithm produces a 128-bit hash with an extremely low collision probability (~1 in 2^128^ for any two distinct sequences), identical hash values can be assumed to represent identical CDS. In a typical bacterial genome with ~5,000 CDSs, the probability of any collision is negligible (~7.35×10^−32^). An overview of the CDST pipeline is provided in Fig. S1.

### CDS prediction and hash list construction

Genome assemblies (in FASTA format) were all annotated with Prodigal (v.2.6.3), which predicted CDSs in a uniform manner to maximize cross-sample comparability. The predicted CDS sequences are then filtered to remove those containing ambiguous nucleotides (e.g. ‘N’). Predicted CDSs were additionally filtered by length. All analyses used CDSs filtered by length ≥201 bp unless otherwise stated. This threshold is widely adopted in gene-by-gene schema construction to mitigate annotation noise from very short ORFs and contig-break artefacts, thereby enhancing locus stability and inter-sample comparability [8,10]. Comparisons to the unfiltered variant appear only in sensitivity checks. For each remaining CDS sequence, an MD5 hash is computed to generate a unique hash value. These hash values are collected into a list for each sample, and all sample hash lists are stored in a JSON database along with the corresponding file names.

### Distance estimation and normalization

Pairwise evolutionary distances between samples are estimated in CDST based on the overlap of CDS hash lists. For a given sample i, let $H_{i}$ denote the set of MD5 hash values corresponding to its predicted CDSs, and let $\left|H_{i}\right|$ represent the total number of CDSs.

For any two samples i and j, the number of shared CDSs is given by the cardinality of their set intersection, $\left|H{}_{i}\cap H_{j}\right|$. The absolute distance from sample i to sample j, denoted as $d_{ij}^{abs}$, is defined as:


$$d_{ij}^{abs}=\left|H_{i}\right|-|H{}_{i}\cap H_{j}|$$

The absolute distance matrix $D^{abs}=\left(d_{ij}^{abs}\right)$ is thus constructed. Since the total number of CDSs may vary across samples, this matrix is generally asymmetric. To normalize for sample-specific differences in CDS content, a relative distance is computed as:


$$d_{ij}^{rel}=\frac{d_{ij}^{abs}}{\left|H_{i}\right|}=1-\frac{\left|H{}_{i}\cap H_{j}\right|}{\left|H_{i}\right|}$$

The resulting relative distance matrix is denoted as $D^{rel}=\left(d_{ij}^{rel}\right)$, where each entry $d_{ij}^{rel}\in \left[0,1\right]$ represents the proportion of non-shared CDSs from the perspective of the sample i.

To facilitate clustering and graph-based visualization, a symmetric distance matrix is derived by taking the minimum of the directional relative distances:


$$d_{ij}^{sym}=min(d_{ij}^{rel},d_{ji}^{rel})$$

The symmetric matrix $D^{sym}=(d_{ij}^{sym})$ is then used to construct a minimum spanning tree (MST), which provides an interpretable graphical representation of sample relationships. Hierarchical clustering was performed with scikit-learn’s *AgglomerativeClustering* (v.1.5.1) using the precomputed distance matrix $D^{sym}$ and *average* linkage. Flat clusters were defined by cutting the dendrogram at specified CDST thresholds for strain classification and grouping.

### Merging and updating databases

CDST supports merging of multiple pre-constructed hash list JSON databases. If a CSV file containing a pre-computed absolute distance matrix is also provided, the tool can merge these matrices into a new, comprehensive distance table. Additionally, the tool allows for the comparison of new CDS sequence files against an existing JSON database and distance matrix, outputting the closest matching sample(s) and their relative distances. This feature enables continuous updates to an established sample relationship tree.

### Consistency validation

Pairwise sample distances were computed and compared against distances derived from the unfiltered CDST, alongside the conventional cgMLST, wgMLST, cgSNP, Mash and SKA workflows. For the wgMLST workflow, allele profiles were generated using chewBBACA (v.3.3.9) [11] and processed with GrapeTree (v.1.5.0) [12] to compute a pairwise distance matrix, which was then normalized to the [0,1] range via min–max scaling. For cgMLST, the chewBBACA profiles were filtered to retain loci present in at least 95% of samples before distance calculation with GrapeTree. In the cgSNP workflow, genomes were annotated with Prokka (v.1.14.6) [13], the core genome was determined and aligned using Roary (v.3.13.0) [14], recombinant regions were identified and masked using Gubbins (v.2.4.1) [15] and SNP distances were computed with snp-dists (v.0.8.2), followed by natural log transformation and min–max normalization. For the Mash workflow, SourMASH (v.4.8.14) [16] was applied with *k-mer* length of 31 and scaling of 1,000 times, after which the results were log-transformed and normalized using the min–max method. For SKA, pairwise distances were computed using SKA (v.1.0) with the default k-mer length of 15, and the resulting Jaccard indices were transformed into a distance matrix as distance=1-Jaccard [7]. Distance pairs were classified into *concordant* and *discordant* groups using a relative rank difference threshold of 0.05. For each pair, the relative rank difference was calculated as $\left|r_{1}-r_{2}\right|/N_{pairs}$, where $r_{1}$ and $r_{2}$ are the ranks of the pair, assigned in ascending order of distance, in the two distance lists, and $N_{pairs}$ is the total number of pairs compared.

Mobile genetic elements (MGEs) were annotated for all genomes using Mobile Genetic Element Finder (v.1.1.2) [17], and the number of unique MGEs identified per genome was recorded. Correlation analyses were then performed between MGE counts and relative rank differences, and statistical comparisons of MGE abundances between groups were conducted.

### Clustering evaluation

The clustering behaviour of CDST (v.0.2.0) was benchmarked on a reference set of 1,961 *S. enterica* genomes, comprising all RefSeq complete genome assemblies available up to 8 January 2025. The GCF accession numbers of all analysed genomes are provided in Table S1. Five established workflows — cgMLST, wgMLST, cgSNP, SourMASH and SKA — were run on the same genomes for systematic comparison.

For each method, a pairwise distance matrix was generated and subjected to agglomerative hierarchical clustering across a grid of distance thresholds (relative distances 0–1, step=0.001; absolute distances incremented by one unit where applicable). Silhouette score quantified cluster cohesion and separation [18], while adjusted mutual information (AMI) and adjusted Rand index (ARI) were used to assess the concordance of the clustering results with external reference groupings [19]. Clustering was implemented with the *AgglomerativeClustering* class in scikit-learn (v.1.5.1), and all indices were calculated using *silhouette_score*, *adjusted_mutual_info_score* and *adjusted_rand_score* [20].

### Conventional typing methods

For multilocus sequence typing (MLST), the *mlst* tool (v.2.23.0; available at https://github.com/tseemann/mlst) was employed using the *S. enterica* scheme from the PubMLST database [21]. Allele calling and sequence type (ST) assignments were performed locally for all 1,961 genomes. For *in silico* serotyping, *SeqSero2* (v.1.3.1) was used with default parameters to predict *Salmonella* serovars from genome assemblies [22].

### Cross-species validation

Two additional RefSeq datasets were analysed for generalizability validation with the CDST (v.0.2.0) pipeline: 2,546 complete genomes of *Escherichia coli* released between 2021 and 2025 and 2,344 genomes of *Listeria monocytogenes* (scaffold, chromosome and complete levels). All assemblies were processed exactly as for *S. enterica*. The information of all analysed genomes is provided in Table S2.

### Assessment of the effect of input sequence completeness

To assess the impact of input sequence completeness on CDST performance, 1,961 complete *S. enterica* genome assemblies were randomly fragmented into contigs with lengths drawn from a log-normal distribution. To simulate incomplete assemblies, bases were removed from both ends of each contig by$contiglength\times \left(1-coverage\%\right)/2$, generating datasets with target coverages of 70%, 80%, and 90 % to mimic lower-quality sequencing outputs.

Completeness of benchmark genes for each dataset was validated using BUSCO (v.6.0.0) [23]. Three experimental configurations were prepared: (i) a control set comprising complete assemblies; (ii) validation sets comprising assemblies at a single coverage level (50, 60, 70, 80 or 90, representing the corresponding coverage percentages); and (iii) mixed validation sets combining complete assemblies with assemblies of progressively higher coverage levels. The mixed validation sets were designated as Mix≥50, Mix≥60, Mix≥70, Mix≥80 and Mix≥90, corresponding to minimum coverage thresholds of 50%, 60%, 70%, 80% and 90%, respectively, with complete assemblies included in all groups. For each mixed set, the random sampling procedure was repeated three times, and the mean values of all calculated parameters were reported.

For each validation set, pairwise distances generated by CDST were compared with those from the control set using correlation metrics (*R*², Pearson’s *r*, Spearman’s *ρ* and cosine similarity). In addition, the concordance of hierarchical clustering results at three predefined resolution levels was assessed against the control set using ARI and AMI, summarized by their relative area under the curve (AUC) values, across different numbers of clusters (K) thresholds.

### Benchmark environment

Runtime and storage benchmarks were executed on an Intel Xeon Gold 6258R (2.70 GHz) workstation equipped with 512 GB RAM and CentOS Linux release 7.9.2009, using a single core. Full hardware and environment details are provided in Table S3.

## Results

### Evaluation of CDST distance consistency and clustering resolution

**Fig. 2.**
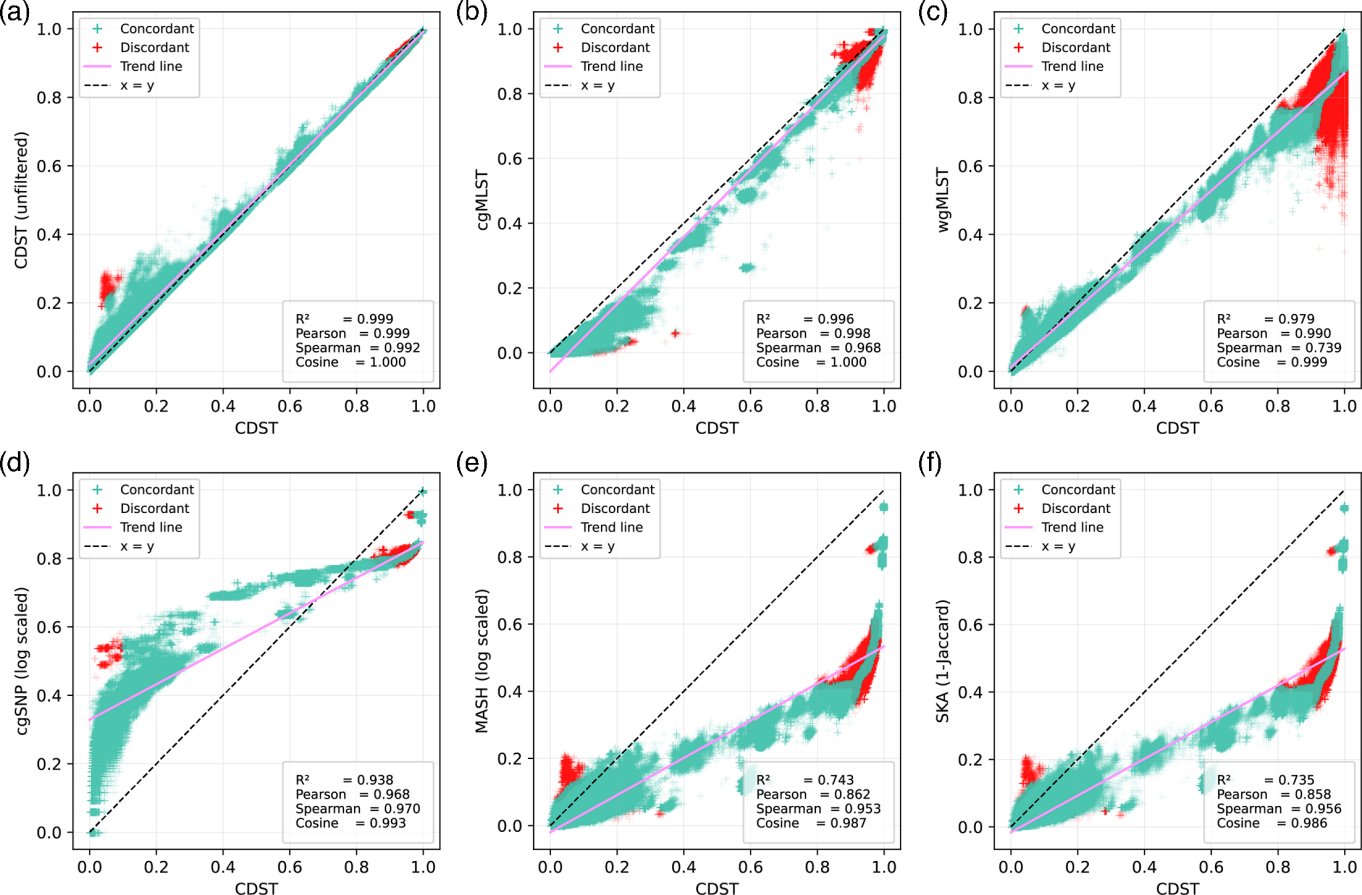
Scatter plots comparing pairwise sample distances for 1,961 *Salmonella* genomes across different methods. Each plot represents the comparison between two methods, with the red solid line showing the linear regression fit and the black dashed line representing the 𝑥 = 𝑦 reference. Panels (a–f) illustrate (a) length-filtered CDST vs. unfiltered CDST, (b) CDST vs. cgMLST, (c) CDST vs. normalized wgMLST, (d) CDST vs. log-scaled cgSNP, (e) CDST vs. log-scaled Mash and (f) CDST vs. SKA distances (Jaccard indices transformed as 1 − Jaccard). *R*² (from linear regression), Pearson’s *r*, Spearman’s *ρ* and cosine similarity are annotated on each plot. Points representing concordant and discordant pairs (defined by a relative rank-difference threshold of 0.05) are shown in different colours.

CDST was applied to the *S. enterica* reference genome set to assess distance concordance with established methods. Scatter plots (Fig. 2) were generated for each pairwise comparison (excluding self-comparisons), and statistical analyses were performed, including linear regression (*R*²), Pearson correlation (*r*), Spearman’s rank correlation coefficient (*ρ*) and cosine similarity. Applying a minimum length filter to the CDSs had only a marginal impact on CDST results. Length-filtered CDST distances showed an almost perfect linear relationship with unfiltered CDST distances (*R*²=0.999, *r*=0.999, cosine similarity=1.000). A slight decrease in rank concordance was observed (*ρ*=0.992), driven by a small subset of pairs at both the most distant and closest ends. These results indicate that applying a length filter exerts minimal overall influence on CDST outcomes, although it can slightly affect the rank order of distances for extreme sample pairs.

The correlation metrics also indicate excellent concordance between CDST and established typing methods. Specifically, the comparison between CDST and cgMLST revealed a nearly perfect linear relationship (*r*=0.998, *R*²=0.996) and rank concordance (*ρ*=0.968). As another gene-by-gene approach, wgMLST also showed strong linear correlation with CDST (*r*=0.990, *R*²=0.979); however, it exhibited the lowest rank concordance among all methods (*ρ*=0.739). By contrast, non-gene-by-gene approaches (cgSNP, Mash and SKA), although lacking strong linear correlation with CDST, consistently displayed high rank concordance (*ρ*=0.970, 0.953 and 0.956, respectively). For context, the comparison between cgMLST and wgMLST (both generated using chewBBACA and GrapeTree) produced a Pearson correlation of 0.988, a Spearman coefficient of 0.742 and an *R*² of 0.977. These results collectively demonstrate that CDST aligns closely with conventional methods, especially for cgMLST.

MGEs can potentially affect the distance concordance of whole-genome gene-by-gene methods [24]. To evaluate whether MGEs influence CDST-derived results, we computed the average relative rank difference for each genome across all pairwise comparisons between CDST and cgSNP. Genomes were subsequently stratified into *concordant* (average rank difference ≤0.05) and *discordant* (>0.05) groups (Fig. S2). A weak but statistically significant monotonic association was observed between MGE counts and relative rank differences (Spearman’s *ρ*=0.1005, *P*=8.25×10^−6^), while the linear fit explained little variance (*R*²=0.047). In the *concordant* group (*n*=1,368), MGE counts averaged 30.68 (median=28, sd=11.70, range=11–114); in the *discordant* group (*n*=593), the mean was 31.86 (median=28, sd=13.45, range=10–87). A Mann–Whitney *U* test indicated no significant difference in MGE abundance between groups (*U*=394,170 *P*=0.320). Taken together, although a weak monotonic association is detected, MGEs do not materially affect the relative rank concordance between CDST and cgSNP and are unlikely to account for the observed discrepancies.

### Comparative evaluation of hierarchical clustering behaviour across typing methods

To systematically compare clustering behaviour across methods, we generated hierarchical clustering profiles for CDST, cgMLST, wgMLST, cgSNP, Mash and SKA and evaluated partitions across the full threshold range using silhouette, AMI and ARI. For each method, we scanned relative thresholds from 0 to 1 in 0.001 increments and absolute thresholds in unit steps, computing silhouette scores and cluster counts (Fig. S3).

Gene-by-gene approaches showed broadly similar resolution characteristics. CDST reached its global silhouette maximum near the lineage/population scale (~0.809 at distance threshold 0.441; 186 clusters), closely mirroring cgMLST (~0.870 at 0.401; 182 clusters) and wgMLST (~0.789 at 0.403; 181 clusters). Together, these gene-by-gene methods converged on ~186–188 clusters at or near their optima and aligned strongly with CDST within the 0.416–0.464 concordance window (Table 1).

**Table 1.** Summary of clustering quality and consistency between CDST and alternative genome typing methods

Method	Threshold type	Peak silhouette score (threshold, #cluster)	Best AMI w/ CDST (threshold, #cluster)	Best ARI w/ CDST (threshold, #cluster)
**CDST**	Relative distance	0.809 (0.441,186)	–	–
**cgMLST**	Absolute distance	0.852 (1147, 167)	0.993 (0.464, 186)	0.999 (0.416, 188)
	Relative distance	0.870 (0.401,182)	0.999 (0.464, 186)	1.000 (0.416, 188)
**wgMLST**	Normalized relative distance	0.789 (0.403, 181)	0.999 (0.464, 186)	1.000 (0.464, 186)
**cgSNP**	Absolute distance	0.924 (3380, 184)	0.992 (0.404, 191)	0.986 (0.404, 191)
	Log-normalized relative distance	0.494 (0.693, 183)	0.992 (0.404, 191)	0.986 (0.404, 191)
**Mash**	Relative distance	0.756 (0.224, 180)	0.992 (0.588, 167)	0.998 (0.588, 167)
**SKA**	1-Jaccard distance	0.750 (0.343, 135)	0.995 (0.736, 149)	0.997 (0.736, 149)
	SNP distance	0.043 (0.914, 183)	0.992 (0.414, 189)	0.998 (0.588, 167)

Non-gene-by-gene methods exhibited greater topological heterogeneity yet still yielded highly concordant partitions when aligned to CDST. Using absolute SNP counts, cgSNP achieved the highest overall silhouette (~0.924 at 3380 SNPs; 184 clusters), whereas its log-transformed variant and Mash peaked lower (~0.494 and ~0.756). SKA followed the same pattern: with the 1-Jaccard distance, the silhouette peak was moderate (~0.750 at threshold 0.343; 135 clusters), but the best agreement with CDST occurred at a slightly coarser partition (149 clusters; AMI 0.995, ARI 0.997); under the SKA SNP-distance formulation, the silhouette maximum was low (~0.043), yet alignments near the CDST concordance window still produced strong agreement (AMI 0.992 at 189 clusters; ARI 0.998 at 167 clusters) (Fig. S4).

Taken together, gene-by-gene methods (CDST, cgMLST and wgMLST) displayed higher and closely matched silhouette optima near 186–188 clusters, while non-gene-by-gene methods (cgSNP, Mash and SKA) varied in internal silhouette heights and optima. Among all schemes, the CDST partition at threshold 0.441 (*HC441*) balanced strong internal cohesion with high cross-method agreement, representing a robust and biologically meaningful resolution for downstream analyses.

### Evaluation of CDST thresholds for multiscale genome-based clustering

Based on cgMLST absolute allele difference distances calculated from 1,961 *S. enterica* genomes, a local maximum in silhouette score (0.582) was observed at a clustering threshold of 25 allele differences, corresponding to 598 clusters. This peak was the highest within the range of 0 to 33 allele differences, indicating an optimal balance between intra-cluster cohesion and inter-cluster separation.

Previous outbreak studies have consistently reported that epidemiologically linked isolates differ by cgMLST (allelic difference ≤ 25), supporting the operational use of this threshold for outbreak-level classification [24,25]. Accordingly, a threshold of ≤ 25 allelic differences by cgMLST was used as the reference standard for outbreak-related clustering and is applied throughout this study unless specified otherwise.

**Fig. 3.**
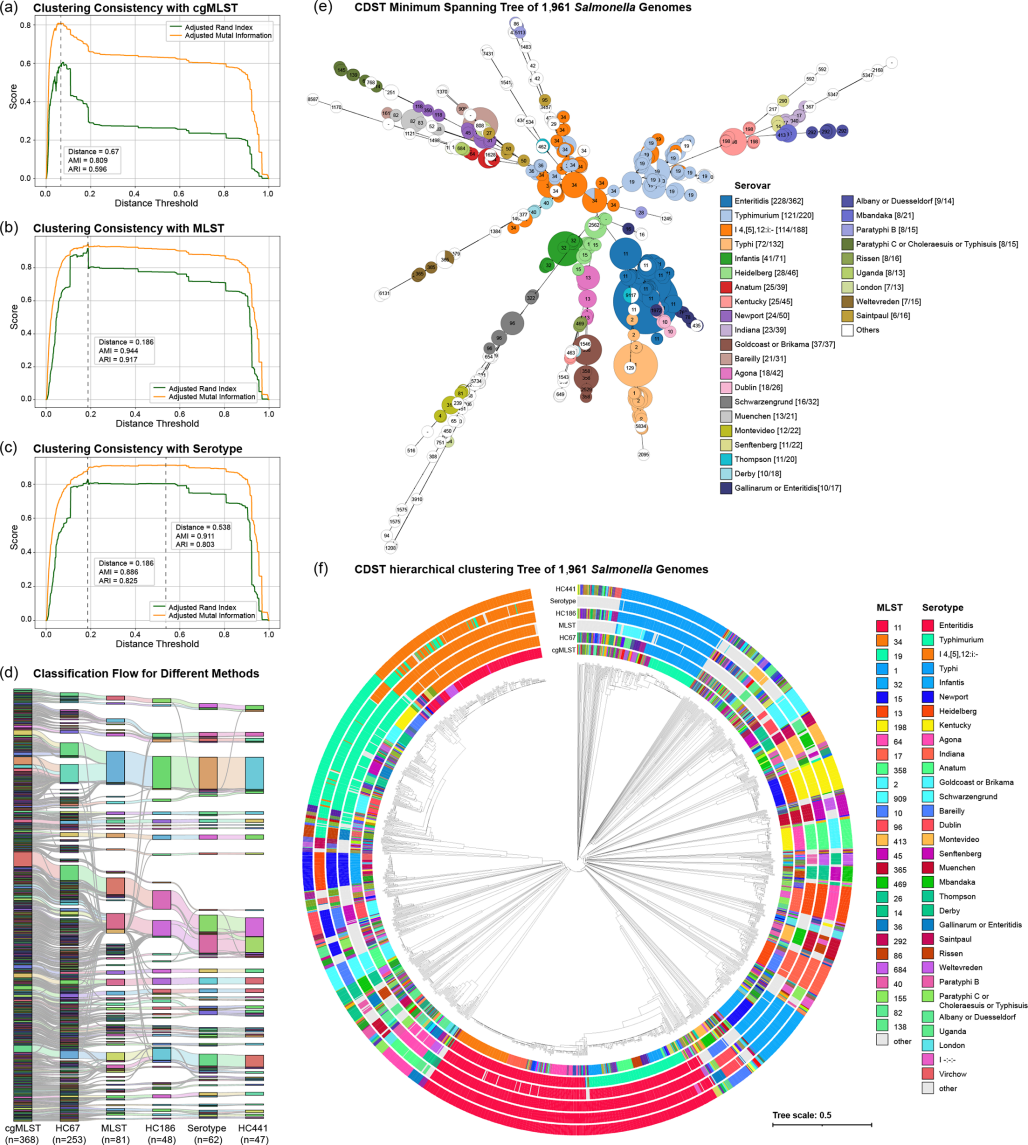
Multi-resolution clustering concordance of CDST compared with established classification frameworks. (a–c) AMI and ARI between CDST and (a) cgMLST, (b) MLST and (c) predicted serotypes across CDST thresholds (0–1, step=0.001). Vertical dashed lines mark peak AMI/ARI values. (d) Sankey diagram showing genome classification transitions across six schemes (cgMLST, *HC67*, MLST, *HC186*, serotype and *HC441*). Only *HC441* clusters with ≥5 isolates were retained; category counts are indicated below each axis. (e) Minimum-spanning tree of 1,961 *S. enterica* genomes based on CDST distances, with genomes <0.01 distance merged. Node size reflects merged sample counts; colours indicate major serotypes; numbers denote MLST types. (f) Hierarchical clustering dendrogram (average linkage) based on pairwise CDST distances of the same genomes, with colour strips annotating the six classification schemes.

To identify a corresponding threshold in the CDST distance space, pairwise AMI and ARI were calculated between the cgMLST partition and the hierarchical clustering results derived from the CDST relative distance matrix (thresholds ranging from 0 to 1, incremented by 0.001). The highest concordance by AMI was observed at a CDST distance threshold of 0.067, where the AMI reached 0.809 and the ARI was 0.596 (Fig. 3a). This threshold was designated as *HC67*, representing the outbreak-level clustering resolution under the CDST framework.

To further evaluate the hierarchical clustering behaviour of the CDST framework across broader taxonomic levels, external reference groupings based on MLST and serotyping were used as benchmarks. Among the 1,961 *Salmonella* genomes analysed, MLST profiles were successfully annotated for 1,897 isolates, yielding 237 distinct STs. Serotype predictions identified 171 unique serotypes across the same dataset.

For MLST-based classification, both AMI and ARI achieved their maximum values at a CDST threshold of 0.186, reaching 0.944 and 0.917, respectively, indicating excellent concordance between CDST clusters and established sequence types (Fig. 3b).

For serotype-based classification, the AMI peaked at a higher threshold of 0.538 (0.911), while the ARI reached its maximum at 0.186 (0.825). Although the AMI at 0.186 (0.886) was slightly lower than the peak value, the ARI curve exhibited a distinct local maximum at this threshold, whereas the AMI curve remained relatively smooth (Fig. 3c). This suggests that the clustering configuration at 0.186 provides a more discrete partition that better reflects serotype-defined boundaries.

Based on the simultaneous optimization of both AMI and ARI for MLST, as well as the local ARI maximum and generally high AMI for serotype classification, the threshold of 0.186 (termed *HC186*) was selected as the representative resolution for lineage- or serotype-level clustering in the CDST framework.

Complete sample-to-cluster assignments for all six schemes are provided in Table S1. After excluding sparse categories, 1,681 genomes from *HC441* clusters with at least five isolates were retained. Concordance across resolutions is illustrated by the Sankey diagram (Fig. 3d), showing strong agreement between neighbouring levels (e.g. cgMLST and *HC67*), whereas broader groupings such as serotype and *HC441* encompass multiple fine-grained clusters.

To further clarify the two complementary ways in which CDST represents genomic relationships, we provide both graph-based and tree-based visualizations within the framework. The MST (Fig. 3e) preserves the local topology implied by CDST distances: very short edges delineate nearly identical genomes and emphasize densely connected cliques, while node sizes highlight the expansion of common STs and serotypes after merging genomes with CDST distances <0.01. In contrast, the complete hierarchical clustering dendrogram (Fig. 3f) presents the global tree structure from which threshold-based partitions are derived. Coloured strips annotate the six classification schemes, demonstrating how the three CDST levels (*HC67, HC186* and *HC441*) are embedded in a single hierarchy and broadly aligned with cgMLST, MLST and serotype boundaries. Together, these complementary views show that CDST supports both MST-based exploratory analyses and hierarchical, threshold-based clustering, providing flexible resolution suitable for multiscale applications in epidemiology, population genetics or evolutionary genomics.

### Evaluation of CDST clustering performance and representativeness across thresholds

**Fig. 4.**
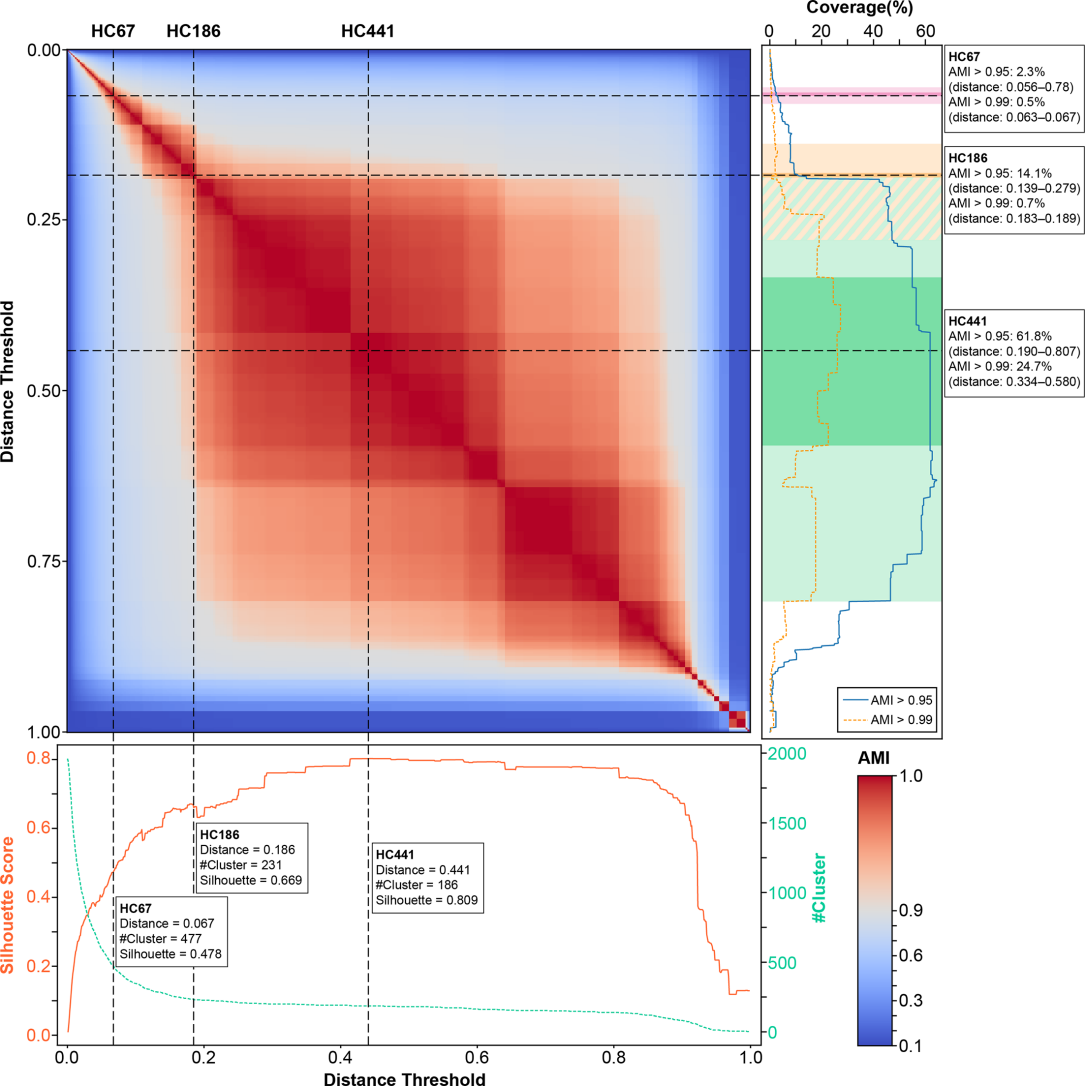
Clustering behaviour of CDST across distance thresholds assessed by AMI, silhouette score, cluster count and coverage**.** The heatmap displays pairwise AMI values between hierarchical clustering results obtained at CDST distance thresholds from 0 to 1, incremented by 0.001. At each threshold, agglomerative hierarchical clustering was performed using the distance as the cutoff. The lower panel shows the corresponding cluster counts and silhouette scores. The right panel depicts the proportion of samples included in high-confidence partitions (AMI ≥0.95 and ≥0.99). Key thresholds *HC67*, *HC186* and *HC441* are highlighted across all panels. Their corresponding cluster counts, silhouette scores and coverage percentages (AMI ≥0.95 and ≥0.99) are specifically annotated. In the coverage panel, shaded regions indicate the distance intervals where each threshold satisfies the specified AMI criteria.

To assess the performance and generalizability of CDST clustering across different resolutions, a comprehensive analysis was performed based on three complementary metrics: silhouette score, number of clusters and clustering consistency evaluated by AMI. High-confidence partitions were defined as clusters exhibiting pairwise AMI values greater than 0.95 or 0.99. The overall clustering behaviour across the full range of CDST distance thresholds (0 to 1, step=0.001) is illustrated in Fig. 4.

Three previously defined thresholds, *HC67*, *HC186* and *HC441*, were selected for representative evaluation of clustering quality and dataset-level consistency. At *HC67*, 477 clusters were identified with a silhouette score of 0.478. The AMI ≥0.95 coverage was 2.3%, and the AMI ≥0.99 coverage was only 0.5%. This threshold coincides closely with the AMI peak of cgMLST but is within the phase of steep rise in the CDST silhouette coefficient as the distance threshold increases, indicating limited representativeness and stability at lower thresholds when used for high-resolution tracing and clustering. At *HC186*, 231 clusters were observed with a higher silhouette score of 0.669. The AMI >0.95 coverage was 14.1%, while the AMI ≥0.99 coverage was 0.7%. The ARI curve for serotype classification showed a local maximum at this threshold (Fig. 3c), while the AMI curve remained relatively smooth, suggesting that although *HC186* achieves strong internal clustering, its external representativeness is constrained to a narrow subset of the dataset. At *HC441*, the number of clusters was reduced to 186, while the silhouette score reached its global maximum of 0.809. The AMI ≥0.95 and ≥0.99 coverage values were 61.8 and 24.7 %, respectively, the highest among all examined thresholds. This indicates that clustering at *HC441* is not only cohesive but also consistent across a large proportion of the dataset.

Notably, the AMI ≥0.95 coverage curve remained below 5% throughout the distance range <0.189 and exhibited a sharp increase beginning around 0.190. This inflection point indicates a transition from threshold-sensitive clustering behaviour to more stable and broadly representative partitions.

### Cross-species validation

**Fig. 5.**
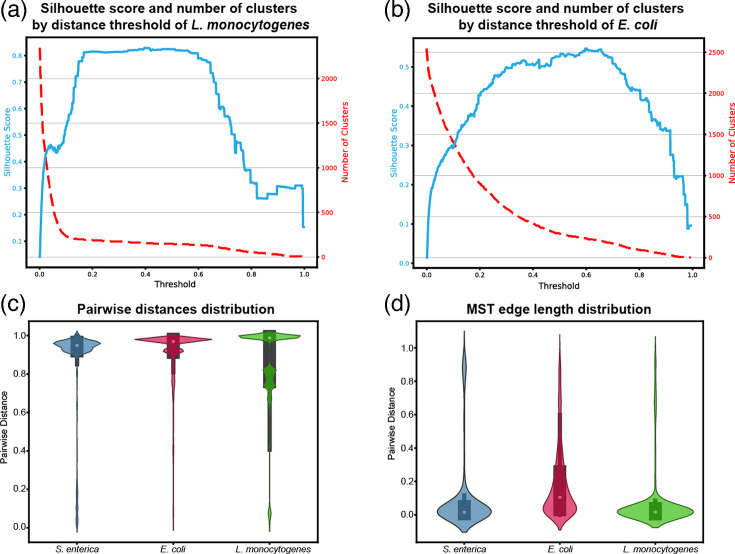
Clustering characteristics and distance distributions across bacterial species. (a) Silhouette score and corresponding number of clusters across distance thresholds for *L. monocytogenes*. (b) Silhouette score and corresponding number of clusters across distance thresholds for *E. coli*. (c) Pairwise distance distributions of *E. coli*, *S. enterica* and *L. monocytogenes* are represented by violin plots with embedded boxplots. (d) MST edge-length distributions of the three species are represented by violin plots with embedded boxplots.

CDST was further applied to 2,546 *E. coli* complete genomes and 2,344 *L. monocytogenes* assemblies. The resulting silhouette score curves revealed clear differences in population structure. For *L. monocytogenes*, the silhouette score curve exhibited two distinct plateaus at distance thresholds of 0.031–0.090 (silhouette score 0.433–0.472) and 0.167–0.589 (silhouette score 0.808–0.829), and the global maximum silhouette value of 0.829 occurred at a threshold of 0.397, resulting in 158 clusters (Fig. 5a). This double-plateau pattern of the silhouette coefficient is more pronounced than that observed in the 1961 *Salmonella* genomes. In contrast, *E. coli* showed no distinct plateau; its silhouette curve peaked at 0.547 at a distance threshold of 0.602, yielding 231 clusters, consistent with the species’ broader and more continuous genomic diversity (Fig. 5b).

The pairwise distance distributions exhibited species-specific patterns. The mean pairwise distance was 0.899 (median=0.969, sd=0.162) for *E. coli*, 0.866 (median=0.949, sd=0.242) for *Salmonella* and 0.846 (median=0.988, sd=0.221) for *L. monocytogenes. E. coli* distances were predominantly concentrated in the 0.9–1.0 range, whereas *Salmonella* distances also showed an additional minor distribution between 0.0 and 0.2. In contrast, *L. monocytogenes* distances were more dispersed, with distinct clusters in the ranges of 0.0–0.2, 0.6–0.8 and 0.8–0.9 (Fig. 5c). These results indicate heterogeneous pairwise distance distributions across species, consistent with their differing genomic diversity profiles.

The MST edge length distributions followed the same trend. The mean pairwise edge length was 0.071 (median=0.017, sd=0.180) for *L. monocytogenes*, 0.105 (median=0.016, sd=0.236) for *Salmonella* and 0.186 (median=0.105, sd=0.216) for *E. coli*. All pairwise Welch’s t-tests indicated statistically significant differences between species (*P*<0.01) (Fig. 5d). These results indicate that CDST captures distinct clustering patterns across species with different levels of genomic diversity.

### Impact of assembly completeness on CDST performance

The impact of genome assembly completeness on CDST performance was evaluated by comparing CDST-derived distance matrices and hierarchical clustering from 1,961 complete *S. enterica* genomes against those from simulated assemblies at reduced completeness. Validation sets comprised (i) single-completeness datasets with target coverages of 50–90% in 10% increments and (ii) mixed datasets combining complete assemblies with assemblies meeting minimum completeness thresholds (≥50%, ≥60%, ≥70%, ≥80% and ≥90%). Assembly completeness of the simulated datasets was validated with BUSCO (v.6.0.0). The observed mean±sd values were 51.3±11.5 %, 61.1±10.5 %, 70.7±9.4%, 80.2±8.0% and 89.3±5.8% for the 50–90% sets, respectively, confirming that the simulations closely matched the intended completeness levels.

**Fig. 6.**
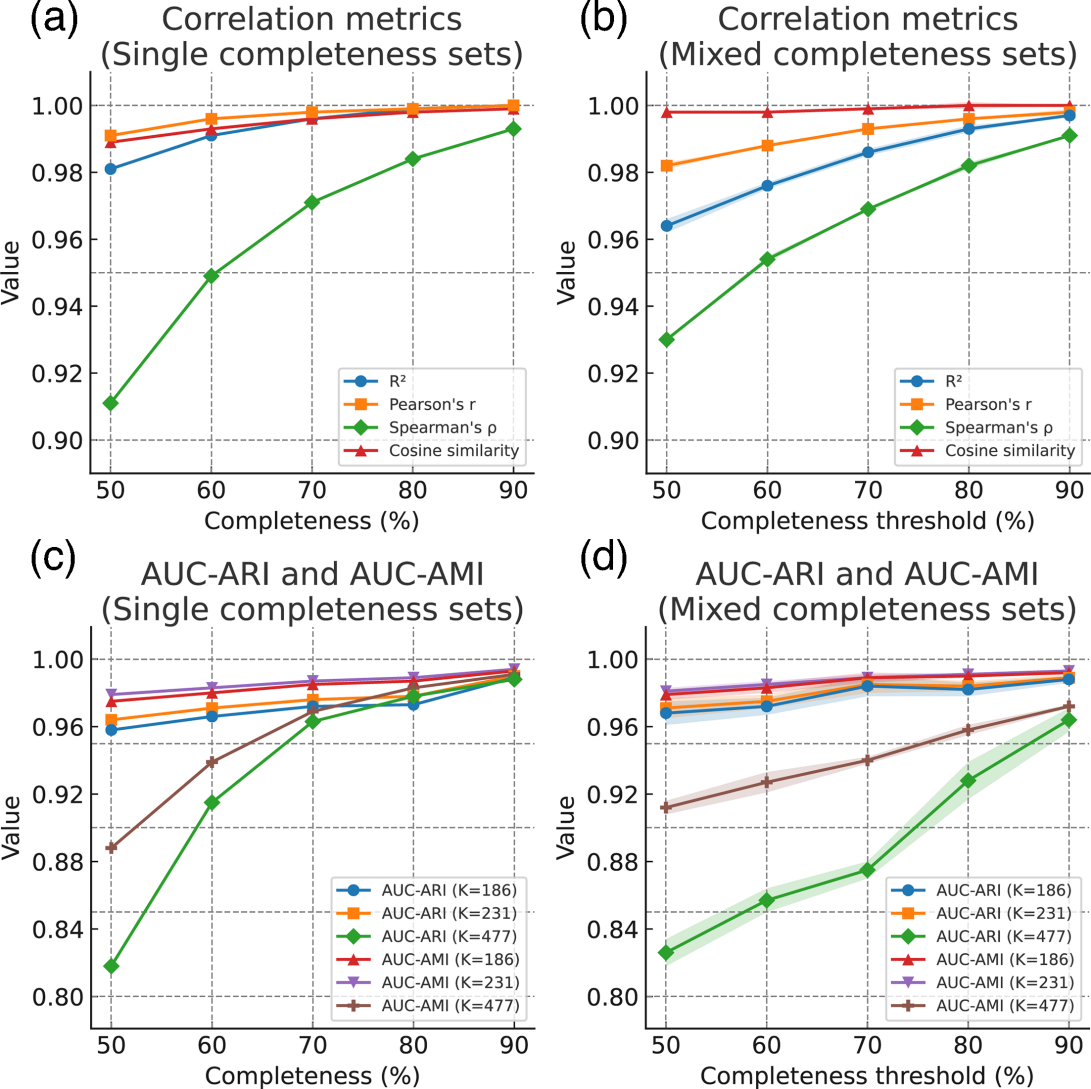
Association of assembly completeness with pairwise distance concordance and clustering consistency in CDST. (a) Correlation metrics (*R*², Pearson’s *r*, Spearman’s *ρ* and cosine similarity) for sets at varying completeness levels. (b) Correlation metrics for mixed completeness sets comprising assemblies with completeness at or above the indicated thresholds; shaded areas represent standard deviations from three replicates. (c) Clustering concordance measured by relative AUC of ARI and AUC of AMI at three hierarchical levels (*K*=186, *K*=231 and *K*=477, corresponding to **HC441**, **HC186** and **HC67**, respectively) for sets at varying completeness levels. (d) Clustering concordance (relative AUC of ARI and AUC of AMI) for mixed completeness sets comprising assemblies with completeness at or above the indicated thresholds; shaded areas represent sd.

Overall, pairwise distance concordance decreased progressively with lower completeness, yet all concordance metrics remained ≥0.90 across all datasets. Among the correlation measures, rank correlation (Spearman’s *ρ*) was most strongly affected (Fig. 6a). Nevertheless, when assemblies of single completeness sets were ≥70% or when mixed completeness thresholds were ≥60 %, Spearman’s *ρ* remained above 0.95, indicating a high level of concordance in the relative ranking of distances (Fig. 6b).

Evaluation of clustering stability further demonstrated that the effect of completeness varied across resolution levels. At broader clustering levels (*K*=231 and *K*=186, corresponding to *HC441* and *HC186*), both single completeness sets ≥50% and mixed completeness sets achieved robust consistency (AUC-ARI and AUC-AMI >0.95, Fig. 6c–d). By contrast, at the higher-resolution level (*K*=447, corresponding to *HC64*), decreases in completeness had a stronger effect on clustering consistency, particularly for mixed datasets. For single completeness sets, assemblies with ≥60 % completeness still achieved AUC-ARI ≥0.90 at *K*=447, which can be considered ‘excellent’ agreement, according to Brusco, M.J. [26]. In contrast, for mixed completeness sets, the AUC-ARI only reached ≥0.90 when the completeness threshold was ≥80%.

Taken together, these findings indicate that the effect of assembly completeness on CDST outcomes is most pronounced for low-completeness assemblies analysed at fine-grained clustering levels, where reductions in consistency become evident. Nonetheless, even for mixed completeness sets at the 50% threshold, ARI values remained above 0.80, which can still be interpreted as ‘good’ clustering agreement [26]. Given that genome assemblies from next-generation sequencing often exhibit variable completeness, we recommend prioritizing assemblies with completeness ≥80% for CDST analysis when high-resolution clustering is required. However, even assemblies with lower completeness can still provide useful clustering information under the CDST framework.

### Computational efficiency

To evaluate computational time and storage requirements, a subset of 100 *Salmonella* genomes was randomly selected from the full dataset of 1961 genomes. All analyses were performed in a single-threaded environment starting from genome assembly files. Benchmark hardware and system environment are described in Table S3.

For the evaluation performed on 100 *Salmonella* genomes, the most time-consuming step in the CDST workflow was the CDS prediction using Prodigal (v.2.6.3), which took ~819 s in total. The subsequent processing steps from CDS extraction to minimum spanning tree construction required only 28 s overall. In comparison, the complete *de novo* ChewBBACA (v.3.3.9) and GrapeTree (v.1.5.0) pipeline took around 6,993 s for the wgMLST workflow and ~7,221 s for the cgMLST workflow for the same set of samples. Moreover, regarding storage efficiency, the original FASTA files occupied 483,188 KB, whereas the hash list JSON files generated by CDST occupied only about 19,458 KB, 4.0% of the original size.

## Discussion

The results of this study demonstrate that the CDST method provides a fast, simple and fully decentralized solution for genome-based typing of bacteria, as exemplified by its high concordance with established methods in a comprehensive dataset of *S. enterica* complete genomes. By avoiding locus-based allele calling and instead using MD5 hash values to represent CDS content, CDST overcomes key limitations of centralized and distributed frameworks such as cgMLST and hash-cgMLST, including dependency on reference databases, storage overhead and data sharing constraints. The near-perfect linear agreement between CDST and cgMLST confirms the method’s consistency and underlines its suitability as a practical alternative to locus-based typing workflows.

Although the overall concordance with cgMLST is high, the choice of clustering threshold remains critical and context-dependent. As genomic surveillance and outbreak response demand different resolution levels, no single fixed threshold is universally applicable. To address this, clustering consistency and external agreement were systematically assessed across distance thresholds using unsupervised evaluation metrics, including silhouette score, AMI and ARI. This framework enabled the comparison of CDST with multiple typing strategies and provided an objective approach for selecting biologically meaningful and technically robust clustering thresholds. An inherent limitation is the dependence on accurate CDS prediction; fragmented draft assemblies or annotation errors could inflate hash distances. Nevertheless, our analyses demonstrate that CDST is broadly robust to incomplete assemblies and maintains reliable performance even under reduced input quality. Importantly, for high-resolution clustering applications, assemblies with at least 80% completeness are sufficient to ensure stable and accurate results.

As a decentralized clustering method, CDST does not inherently provide taxonomic labels across species. Nevertheless, our benchmarking analyses indicate that by systematically traversing the CDST hierarchy and assessing the concordance between clustering results obtained under varying threshold levels and a unified reference classification, it is possible to identify the most appropriate hierarchical resolution of CDST and to establish reliable mappings between CDST-derived clusters and established taxonomic groups. Among the three proposed thresholds of the benchmark of 1,961 *S. enterica*, *HC186* demonstrated the highest AMI and ARI with traditional MLST and serotyping classifications, confirming the utility of CDST as a flexible framework capable of aligning with conventional taxonomic and epidemiological schemes through unsupervised distance-based learning. This capability could be further enhanced in the future by constructing distributable reference frameworks that map known nomenclatures onto optimal CDST thresholds.

In addition to its concordance with external methods, internal clustering quality and dataset-wide consistency were evaluated across thresholds. The comprehensive analysis revealed that *HC441* not only yielded the global maximum silhouette score but also achieved the highest coverage of high-confidence partitions. This suggests that *HC441* represents a stable and informative clustering resolution, suitable for applications such as global population structure analysis or lineage tracking.

A well-recognized challenge in outbreak investigation is the difficulty in establishing a universal threshold for cluster definition using cgMLST. Previous studies have shown that slight differences in allele difference cut-offs (e.g. between 5 and 25 alleles) can result in significant changes in clustering outcomes, making it difficult to standardize outbreak-level classification across datasets and surveillance systems [24,25]. This issue is equally evident in the CDST framework. Within the low-distance range (distance <0.190), clustering results were highly sensitive to threshold selection, with AMI-based coverage metrics showing abrupt fluctuations. Such instability underscores the challenge of defining consistent outbreak-level groupings using fine-scale genomic distance metrics.

Using the *S. enterica* serovar Agona collection from Alikhan *et al.* [1], we re-examined outbreak-level topology to assess the applicability of CDST for source tracing and to probe potential effects of MGEs on fine-scale clustering. Of the 1,082 isolates described by Alikhan *et al*. 774 were retrievable from GenBank and were used to construct a CDST-based MST (Fig. S5A). We compared this graph with the cgMLST- and wgMLST-derived MSTs reported by Alikhan *et al*. (Fig. S5B-C) and with an SNP-based phylogeny for 73 Agona isolates reported by Zhou *et al.* [27] [27] (Fig. S5D). Previous work revealed independent acquisitions of phage elements in two historically investigated outbreak groups – group A (Ireland, 2005) and group D (Ireland and other European countries, 2008/2009). As a consequence, wgMLST represented group A and group D as closer related sub-branches than cgMLST and core-genome SNPs (see Fig. 4 in Alikhan *et al*.). In our re-analysis, CDST similarly places group A and group D closer together, consistent with signals influenced by accessory gene content. These observations indicate that, although our benchmarking suggested only a modest overall impact of MGEs on CDST distances, accessory genome changes can still reorder local topology at outbreak resolution.

The cross-species analysis shows that CDST yields coherent, hierarchically structured distance landscapes across taxa with distinct biology and epidemiological contexts. *L. monocytogenes* exhibits a clear double-plateau silhouette pattern, including a pronounced low-distance stability interval consistent with outbreak-scale thresholds; *S. enterica* displays an intermediate pattern with a silhouette plateau at distance thresholds that approximate serotype partitions; and *E. coli* shows a single broad optimum and longer MST edges, consistent with greater genomic diversity and limited outbreak-driven clustering. Part of these contrasts likely reflects differences in sampling frames: the *E. coli* set was restricted to recent complete genomes (fewer near-identical, outbreak-linked isolates), the *L. monocytogenes* set spanned assembly levels and was plausibly enriched for public-health submissions, and *S. enterica* is often curated and analysed in serotype-specific cohorts. Practically, these properties argue that clustering thresholds should be calibrated to each population’s diversity and that CDST can serve as a lightweight, hypothesis-generating screen for population-genetic signals, where stability intervals in silhouette space and global pairwise distance or MST edge-length distributions can flag potential outbreak enrichment or discrete lineages/subspecies.

## Conclusion

An MD5 hash-based coding sequence typing method for bacterial genomic analysis, termed CDST, was developed. Bypassing allele annotation and directly comparing CDS hash lists, CDST offers improved performance and enhanced data privacy over cgMLST, wgMLST, cgSNP and Mash. Validation on a large *Salmonella* dataset showed that CDST distances are highly consistent with established typing methods, with Pearson correlation coefficients exceeding 0.99 when compared to cgMLST and wgMLST. This consistency, combined with significantly reduced computation time and storage requirements, makes CDST a promising alternative for rapid and scalable bacterial typing.

CDST offers three key advantages. First, it is decentralized: the method requires no central allele database nor the upload of complete genomes, permitting secure and privacy-preserving strain comparison. Second, it ensures cross-laboratory comparability: MD5 hashing is deterministic across computing environments yet cannot be reverse-engineered; laboratories can, therefore, exchange compact JSON hash lists and still obtain directly comparable distances without revealing raw sequences. Third, it is computationally and storage-efficient: apart from the unavoidable CDS-prediction step, the pipeline reduces processing time and disc usage by an order of magnitude, making it well-suited to large-scale surveillance and outbreak investigation. Although CDST is robust to inputs with varying assembly completeness, to ensure high reliability at any resolution, we recommend using assemblies with ≥80% completeness as inputs.

In addition, three representative clustering thresholds were defined based on benchmarking against existing classification schemes and evaluation using clustering consistency metrics. *HC67* (*distance threshold *=0.067) corresponds to the cgMLST outbreak-level resolution and aligns with the cgMLST (allelic difference ≤ 25) standard; *HC186* (*distance threshold *=0.186) offers the best agreement with MLST and serotype classifications; and *HC441* (*distance threshold *=0.441) represents a robust global clustering level with the highest silhouette score and broad AMI coverage. Together, these levels provide a practical hierarchical framework for multiscale bacterial population structure analysis under the CDST scheme.

## Supplementary material

10.1099/mgen.0.001518 Supplementary Material 1.

10.1099/mgen.0.001518 Supplementary Material 2.

10.1099/mgen.0.001518 Supplementary Material 3.

## Abbreviations

AMI: adjusted mutual information
ANI: average nucleotide identity
ARI: adjusted Rand index
AUC: area under the curve
CDSs: coding sequences
CDST: CoDing Sequence Typer
cgMLST: core-genome multilocus sequence typing
cgSNP: core genome SNP
MD5: Message-Digest Algorithm 5
MGEs: mobile genetic elements
MLST: multilocus sequence typing
MST: minimum spanning tree
SKA: Split Kmer analysis
ST: sequence type
wgMLST: whole-genome multilocus sequence typing
