# Supplementary material for: CoDing Sequence Typer (CDST): a fast, simple, decentralized and interoperable solution for bacterial genomic typing and clustering
Source: Microb Genom. 2025 Oct 23;11(10):001518. doi: 10.1099/mgen.0.001518 (PMC12548756; doi:10.1099/mgen.0.001518)
Supplement: Supplementary Material 1. [file mgen-11-01518-s001.pdf]

Supplementary materials

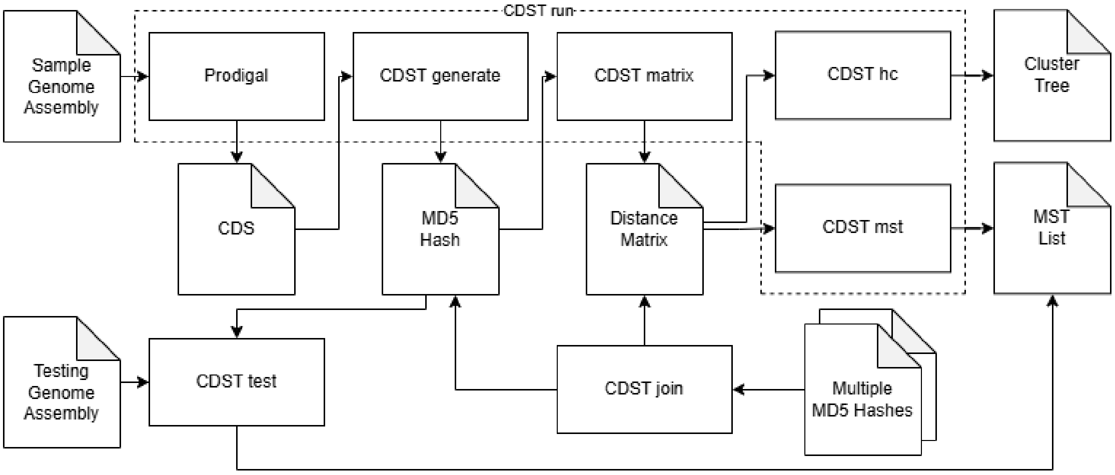

Supplementary Figure S1. An overview of the CDST pipeline

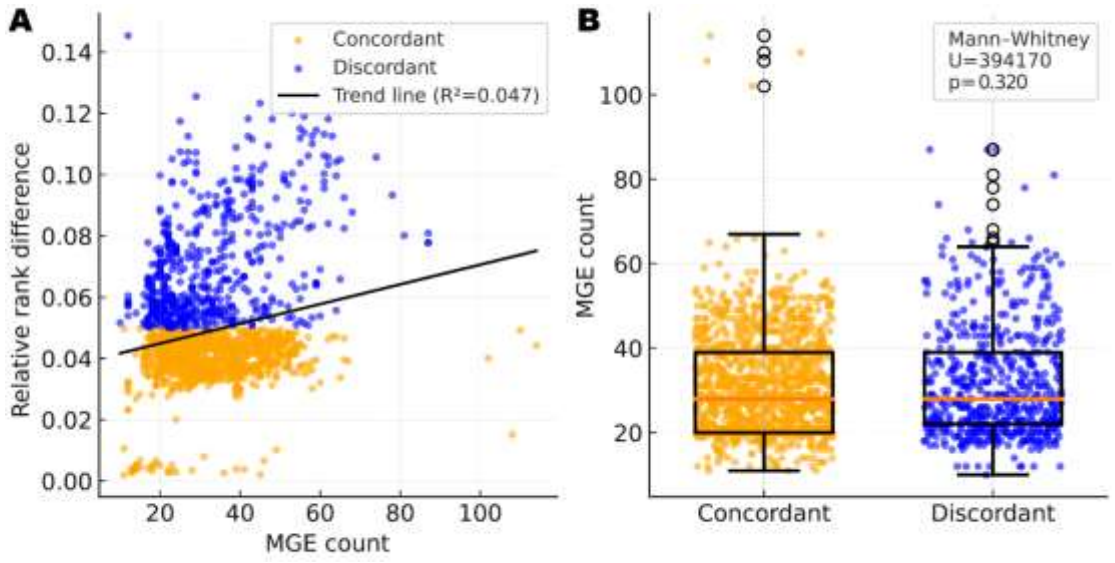

Supplementary Figure S2. Effect of mobile genetic elements (MGEs) on the concordance between CDST- and cgSNP-derived pairwise distances. A. Scatter plot showing the relationship between MGE counts per genome and the average relative rank difference across all pairwise comparisons between CDST and cgSNP. Samples with an average relative rank difference ≥ 0.05 were classified as discordant, whereas those < 0.05 were classified as concordant. **B.** Boxplot of MGE counts for concordant and discordant groups, with individual samples shown as overlaid points.

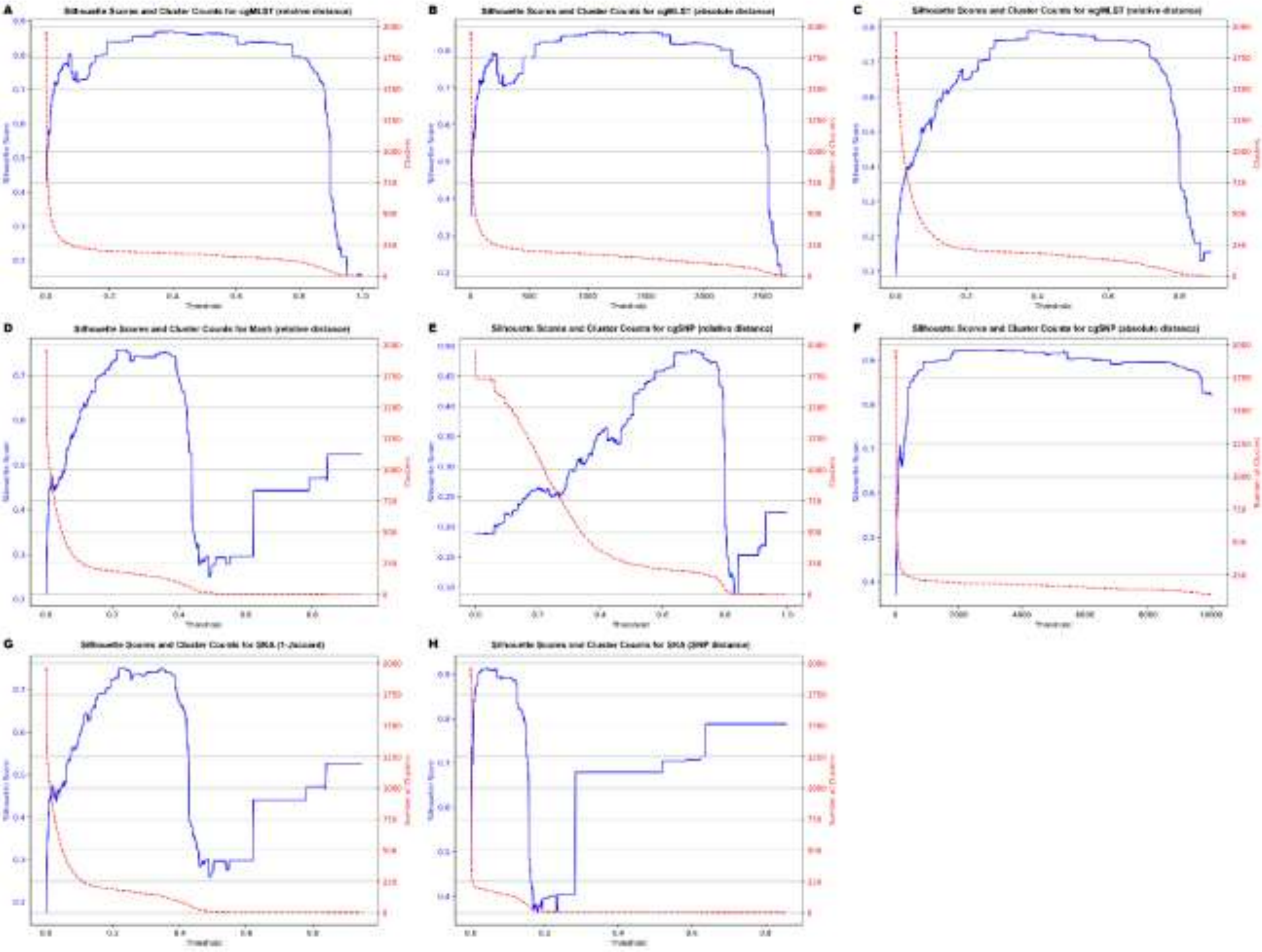

Supplementary Figure S3. Comparison of clustering resolution profiles across genome typing methods. For each method (CDST, cgMLST, wgMLST, cgSNP, Mash), silhouette scores and cluster counts were computed across a full range of distance thresholds. Relative-distance methods were evaluated from 0 to 1 (step = 0.001); absolute-distance methods were evaluated in single-unit steps. Panels **A–H** correspond to CDST, cgMLST (relative/absolute), wgMLST, Mash, cgSNP (log-scaled/absolute), SKA (1-Jaccard), SKA (SNP distance), respectively

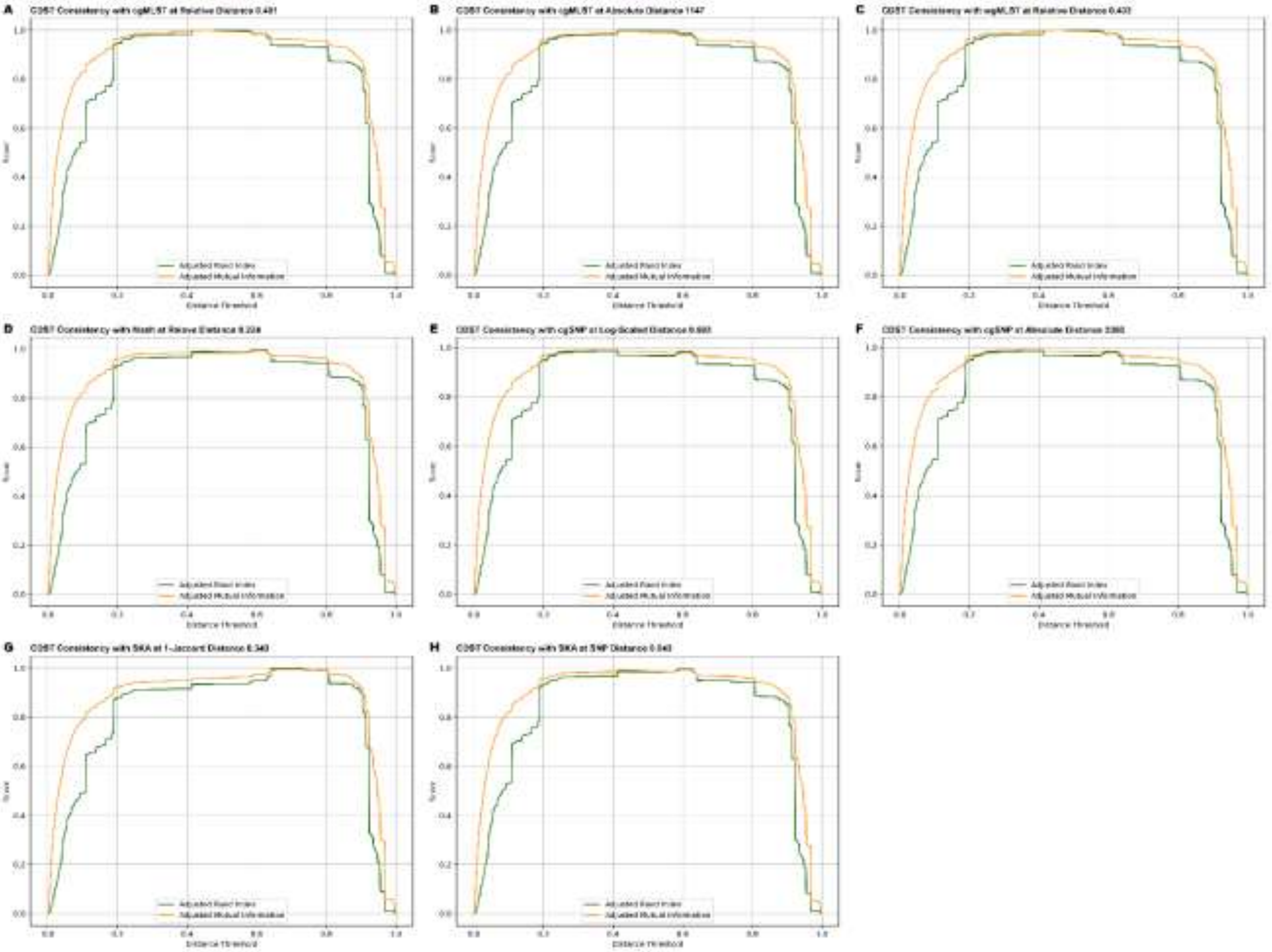

Supplementary Figure S4. Clustering concordance between genome typing methods and the CDST hierarchy. Each panel shows the adjusted mutual information (AMI) and adjusted Rand index (ARI) values comparing the optimal clustering result from a given typing method (based on its peak silhouette threshold) against all hierarchical levels of CDST (distance range 0–1, step = 0.001). High-scoring intervals indicate alignment in clustering granularity between the compared method and specific CDST thresholds. Panels **A–H** correspond to CDST, cgMLST (relative/absolute), wgMLST, Mash, cgSNP (log-scaled/absolute), SKA (1-Jaccard), SKA (SNP distance), respectively, as matched in **Table 1**.

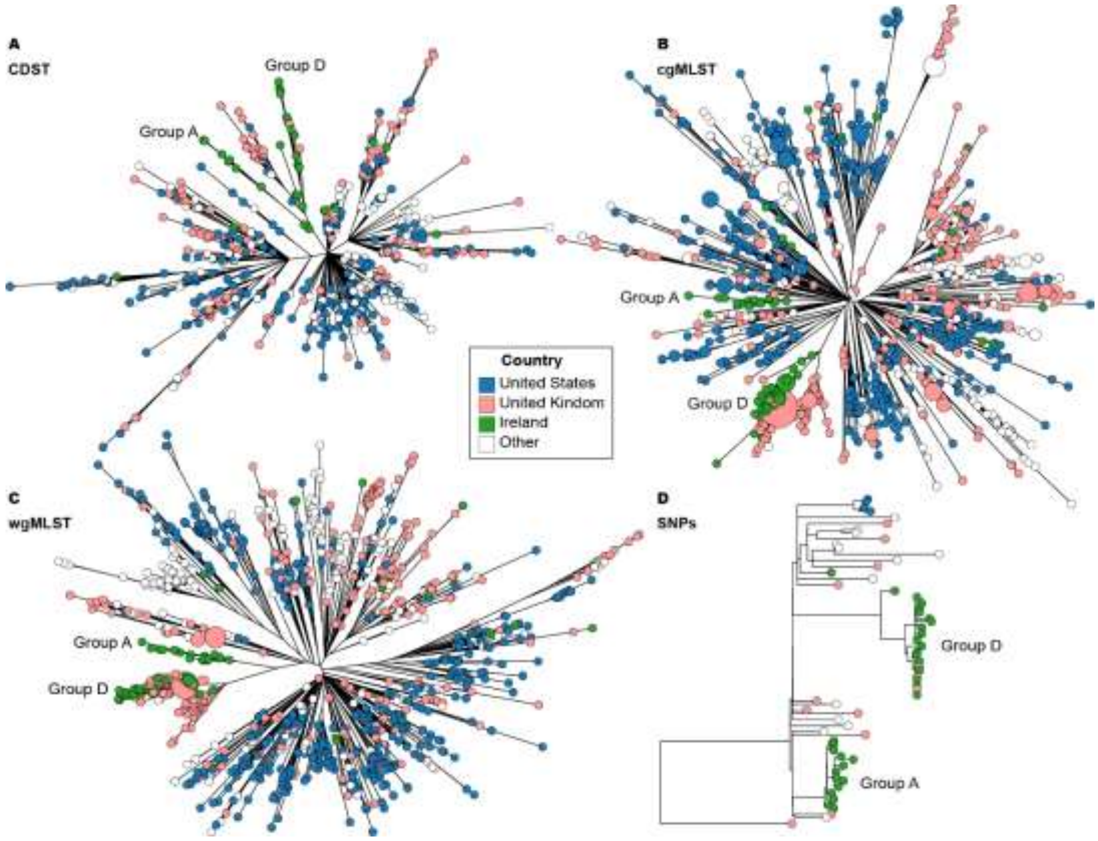

Supplementary Figure S5. Comparison of outbreak-scale relationships in Salmonella enterica serovar Agona. All trees were generated with GrapeTree (v1.5.0). **A.** CDST minimum-spanning tree (MST) for 774 Agona isolates retrievable from GenBank among the 1,082 isolates described by Alikhan et al. (2018). **B.** cgMLST-based MST for the Agona dataset as reported by Alikhan et al. (2018) (MSTreeV2). **C.** wgMLST-based MST for the same dataset (MSTreeV2). **D.** SNP-based phylogeny for 73 Agona isolates reported by Zhou et al. (2013). Node colors indicate the country of isolates.

Supplementary Table S1. Sample-to-cluster assignments for the 1,961 *Salmonella enterica* genomes analysed in this study.

Provided as an individual Excel file (**S1_accessions_clusters.xlsx**) with the following columns: GCF_ACC#, SeroType, MLST, cgMLST, HC67, HC186, HC441.

Supplementary Table S2. Assembly metadata for the 2,546 *Escherichia coli* and 2,344 *Listeria monocytogenes* genomes used in cross-species validation.

Provided as an individual Excel file (S2_accessions_metadata.xlsx) with the following columns: Organism Scientific Name, Organism Common Name, Organism Qualifier, Taxonomy id, Assembly Name, Assembly Accession, Source, Annotation, Level, Contig N50, Size, Submission Date, Gene Count, BioProject, BioSample.

Supplementary Table S3. System Configuration for Performance Evaluation

Component	Specification
CPU	Intel® Xeon® Gold 6258R @ 2.70GHz
Memory	512 GB
Operating System	CentOS Linux release 7.9.2009 (Core)
Python Version	3.12.2
Biopython Version	1.85
Pandas Version	2.2.2
SciPy Version	1.13.1
NetworkX Version	3.3
